# Computer-aided detection (CADe) and diagnosis (CADx) system for lung cancer with likelihood of malignancy

**DOI:** 10.1186/s12938-015-0120-7

**Published:** 2016-01-06

**Authors:** Macedo Firmino, Giovani Angelo, Higor Morais, Marcel R. Dantas, Ricardo Valentim

**Affiliations:** 1 Department of Information and Computer Science, Federal Institute of Rio Grande do Norte (IFRN), Natal, Brazil; 2 Laboratory of Technological Innovation in Healthcare, University Hospital Onofre Lopes (HUOL), Natal, Brazil

**Keywords:** Computer-aided detection system, Lung cancer diagnosis, Medical image analysis, Detection of pulmonary nodules, Likelihood of malignancy, CADe and CADx

## Abstract

**Background:**

CADe and CADx systems for the detection and diagnosis of lung cancer have been important areas of research in recent decades. However, these areas are being worked on separately. CADe systems do not present the radiological characteristics of tumors, and CADx systems do not detect nodules and do not have good levels of automation. As a result, these systems are not yet widely used in clinical settings.

**Methods:**

The purpose of this article is to develop a new system for detection and diagnosis of pulmonary nodules on CT images, grouping them into a single system for the identification and characterization of the nodules to improve the level of automation. The article also presents as contributions: the use of Watershed and Histogram of oriented Gradients (HOG) techniques for distinguishing the possible nodules from other structures and feature extraction for pulmonary nodules, respectively. For the diagnosis, it is based on the likelihood of malignancy allowing more aid in the decision making by the radiologists. A rule-based classifier and Support Vector Machine (SVM) have been used to eliminate false positives.

**Results:**

The database used in this research consisted of 420 cases obtained randomly from LIDC-IDRI. The segmentation method achieved an accuracy of 97 % and the detection system showed a sensitivity of 94.4 % with 7.04 false positives per case. Different types of nodules (isolated, juxtapleural, juxtavascular and ground-glass) with diameters between 3 mm and 30 mm have been detected. For the diagnosis of malignancy our system presented ROC curves with areas of: 0.91 for nodules highly unlikely of being malignant, 0.80 for nodules moderately unlikely of being malignant, 0.72 for nodules with indeterminate malignancy, 0.67 for nodules moderately suspicious of being malignant and 0.83 for nodules highly suspicious of being malignant.

**Conclusions:**

From our preliminary results, we believe that our system is promising for clinical applications assisting radiologists in the detection and diagnosis of lung cancer.

## Background

Lung cancer is a disease characterized by the appearance and uncontrolled proliferation of abnormal lung cells. This disease is one of the main causes of mortality worldwide, with approximately 1.59 million deaths per year [1]. The detection in initial stages is considered the most effective way to improve survival of patients, in which case, the 5-year survival rate is approximately 54 % [2]. On the other hand, when the pathology is detected in advanced stages the survival rate for 5 years is only 4 % [2].

Currently, computed tomography (CT) is the imaging modality most suitable for examinations for early detection of lung cancer. CT provides images with high spatial resolution, high temporal resolution and high resolution of contrast of anatomical structures of the chest. This way, it is possible to display small nodules that could hardly be viewed on conventional radiography [3]. According to Awai et al. [4], the detection rate for lung cancer using CT is 2.6–10 times greater than using analog radiography. However, CT generates a large number of medical images which combined to the workload of radiologists could result in inaccurate detection (failure to detect cancer) or misinterpretation (inability to properly diagnose a tumor). Consequently, computer systems become indispensable to assist radiologists in their decision making.

A CAD (Computer-Aided Detection and Diagnosis) system is a class of computer systems that aim to assist in the detection and/or diagnosis of diseases through a “second opinion” [5]. The goal of CAD systems is to improve the accuracy of radiologists with a reduction of time in the interpretation of images. CAD systems are classified into two groups: Computer-Aided Detection (CADe) systems and Computer-Aided Diagnosis (CADx) systems. CADe are systems geared for the location of lesions in medical images. Moreover, CADx systems perform the characterization of the lesions, for example, the distinction between benign and malignant tumors.

A CADe system for detection of pulmonary nodules usually consists of four main stages: segmentation of the lungs, detection of the candidate nodules, characteristics analysis and elimination of false positives. The segmentation of lung images serves to separate the region in study from other organs and tissues in radiological images. With the segmented images, a search is performed aiming to find abnormal structures present in the lungs, which may be nodules. Then, the characteristics of the possible detected nodules are extracted. The main characteristics that are commonly used for detection of pulmonary nodules are: intensity values of pixels and morphological and texture analysis. Finally, the candidate nodules are classified into nodules or non-nodules by the classifier. This final stage is very important, because it determines the final performance by removing non-nodules and maintaining real nodules.

There are several studies in the literature that propose CADe systems for the detection of pulmonary nodules, among them Armato et al. [6]. They have developed a CADe system that used Linear Discriminant Analysis and had a sensitivity of 70 % with 9.6 FP per case. This system has been validated with 187 nodules (solitary pulmonary nodules and juxtapleural nodules). Suzuki et al. [7] developed a pattern recognition technique based on an artificial neural network for a CADe system, named MTANN, and obtained a sensitivity of 80.3 % with 4.8 FP per case, being tested with 121 nodules (solitary, juxtapleural, juxtavascular and ground-glass nodules). Messay et al. [8] presented a CADe system that used a FLD classifier (Fisher Linear Discriminant) and obtained a sensitivity of 82.66 % with 3 FP per case being validated with 143 nodules (solitary, juxtapleural, juxtavascular and ground-glass nodules).

Tan et al. [9] developed a CADe system that used a neural classifier and obtained a sensitivity of 87.5 % with an average of 4 FP by case, being tested with 574 nodules (solitary, juxtapleural and juxtavascular nodules). Cascio et al. [10] showed an CADe system that made use of a neural classifier and obtained a sensitivity of 97 % with 6.1 FP per case being validated with 148 nodules (solitary and juxtapleural nodules). Teramoto and Fujita [11] proposed a CADe system that used cylindrical filters and SVM and obtained a sensitivity of 80 % with 4.2 FP per case, being validated with 103 nodules (solitary, juxtapleural, juxtavascular and ground-glass nodules). Han et al. [12] used the Hierarchical Vector Quantization (VQ) method and SVM and obtained a sensitivity of 82.7 % with 4 FP per scan, being tested with 490 nodules (solitary, juxtapleural and ground-glass nodules). Erdal and Aybars [13] proposed a CADe system that provides automatic detection of juxtapleural nodule using the GLMR classifier and image processing techniques and obtained an accuracy of 92.91 %, being tested with 124 juxtapleural nodules. They contributed providing seven new features (five shape-based and two both shape and texture based) extracted from nodule candidates that improve the detection performance.

The research showed that 98.6 % of the lung nodules found have remained stable or have become smaller during 2 years of observation, and only 1.4 % of the nodules are actually cancer (malignant structures) [14]. To provide further guidance of malignancy, researchers have been developing systems to aid the diagnosis (CADx).

In general, CADx systems extract the characteristics of the images and use a classifier to measure the malignancy. Usually CADx systems for diagnosis of lung cancer are assessed through the ROC curve, more precisely through the area under the ROC curve ($A_{z}$). A CADx system has been proposed by Shah et al. [15], where they selected 31 characteristics and used logistic regression as classifier, reaching a value of $A_{z}$ 0.92 in distinguishing between 19 malignant nodules and 16 benign nodules, all solitary nodules.

Way et al. [16] developed a CADx system that used morphological characteristics, intensity values and surface characteristics. Using the LDC classifier (Linear Discriminant Classifier), Way et al. obtained a $A_{z}$ of 0.857 on the classification of 124 malignant nodules and 132 benign nodules in 152 patients. Suzuki et al. [17] developed a neural network named MTANN to distinguish between benign and malignant nodules. Suzuki et al. reached a value of 0.88, being tested with 76 malignant nodules and 413 benign nodules. Lee et al. [18] developed a supervised learning system that made use of genetic algorithms and Linear Discriminant Analysis (LDA) for the analysis of 216 characteristics of the images and clinical history of patients. They obtained a $A_z$ of 0.889 when evaluated 62 malignant nodules and 63 benign nodules, all solitary nodules. Orozco et al. [19] used 11 characteristics calculated from the wavelet transform and SVM as classifier. They obtained a $A_z$ of 0.805 being tested with 23 malignant nodules and 22 non-nodules.

CADe and CADx systems for lung cancer have been important areas of research in recent decades. However, these research areas are being worked on separately. According to Fraioli et al. [20] one of the main problems of CADe systems for pulmonary nodules is that they detect the nodules but do not characterize them. Thus, computer systems that only detect nodules are not enough for clinical application. Currently, CADe systems do not present the radiological characteristics of the tumor, resulting in lack of information for radiologists, and CADx systems do not detect tumor and do not have good levels of automation [21]. This way, the new CAD should incorporate into a single software a system for detection (CADe) as well as diagnosis (CADx) [20, 22].

In this article, we propose a new approach to CAD systems encompassing detection of different types of nodules and the determination of the likelihood of malignancy of the nodules through computed tomography scans. Our contribution is to improve the level of automation by performing both detection and diagnosis, with little user intervention, and allowing for a satisfactory accuracy for use in clinical and hospital settings. In addition, we present, for the academic community, the use of the Watershed and HOG techniques for distinguishing the lung structures and feature extraction for pulmonary nodules, respectively. The proposed CAD system and the experimental results of its validation are described below.

## Methods

This section presents the materials used in this research and the proposal of a new CAD system for detection and characterization of pulmonary nodules on CT images.

### Materials

The database used in this research consisted of 420 cases, randomly obtained from LIDC-IDRI (Lung Image Database Consortium) [23]. The LIDC-IDRI database is publicly available in the Cancer Imaging Archive (TCIA), and currently contains 1010 CT scans of the chest, collected in different equipment and different configuration parameters (for example, slice thickness, pixel size and total number of slices). The nodule size ranges from 3 mm to 30 mm and can be primary lung cancer, metastatic disease, benign nodule or indeterminate nature. All nodules were evaluated by four experienced radiologists, that through software tools extracted regions of the nodules and described their likelihood of malignancy. The probability was divided into five degree: highly unlikely, moderately unlikely, indeterminate, moderately suspicious and highly suspicious. More details about the database, such as methods and protocols used to acquire image data and the process of annotation of the lesions can be found in Armato et al. [23].

**Fig. 1:**
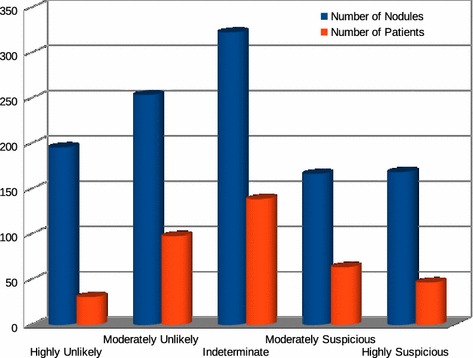
Relationship between each category of malignancy with the number of nodules and the number of patients with those nodules

The nodules obtained in our study were diagnosed by consensus by at least two radiologists. 1109 nodules were used, as follows: 196 highly unlikely of being malignant, 254 moderately unlikely of being malignant, 323 of undetermined malignancy, 167 moderately suspicious of being malignant and 169 highly suspicious of being malignant (as shown in Fig. 1). We use the original DICOM images with 16 bit resolution and evaluations from LIDC-IDRI radiologists for the training and test of the supervised classifier. The database tested consisted of solitary, juxtapleural, juxtavascular, small and ground-glass nodules.

In the research was used exams of 420 patients having between 1 and 8 pulmonary nodules. Among them, 31 with nodules highly unlikely of being malignant, 98 with nodules moderately unlikely of being malignant, 139 with nodules with undetermined malignacy, 64 with nodules moderately suspicious of being malignant and 47 with nodules highly suspicious of being malignant (as shown in Fig. 1).

### Proposed CAD system

The proposed CAD system consists of five stages: 3D segmentation of the lungs in CT images, 3D segmentation of the internal structures of the lungs, detection of candidates nodules, elimination of false positives and the calculation of the likelihood of malignancy. During the entire stage of image processing images with 16 bit resolution were used. Below, the details are presented, step by step.

#### 3D segmentation of lungs

**Fig. 2:**
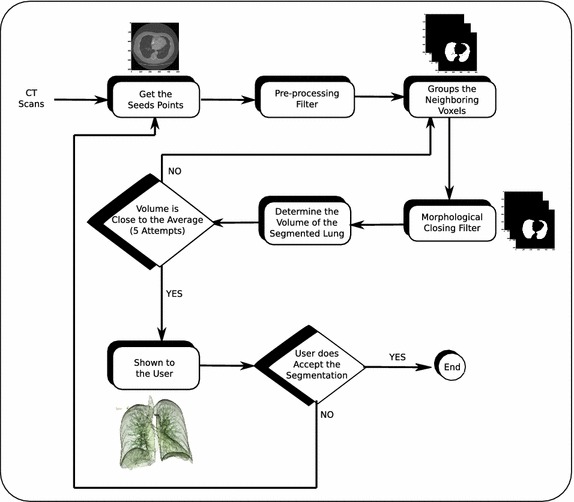
Diagram of the semi-automatic method proposed for the segmentation of lung images in Computed Tomography scans of the chest

The segmentation of the lung images can be defined as a process of delineating the spatial extent of the lungs that appear in images of the thorax. This process is possible in CT images because the attenuation values generated for the image reflect the density of the various tissues. The attenuation is typically expressed as the relative attenuation coefficient, called Hounsfield unit (HU) [24]. Thus, a new semi-automatic method is proposed for segmenting lung CT images combining region growing algorithm and morphological filters, as shown in Fig. 2.

At the beginning of the segmentation process, the user must inform two points (called seeds) in the image that corresponds to pixels that are inside the right and left lung. In sequence, a pre-processing filter is assigned, called Curvature Flow, to eliminate noise in the image. This filter is an algorithm of finite differences proposed by Sethian [25] and implemented by the Insight Segmentation and Registration Toolkit (ITK) [26]. ITK is an open-source, cross-platform system that provides an extensive suite of software tools for image analysis.

Then it uses a segmentation algorithm based on regions growing, called Connected Threshold of the ITK toolkit [26]. This algorithm groups the neighboring voxels according to their intensity within a threshold. As boundaries of similarity, the following values were used: −1000 HU and −200 HU. These values were chosen because they encompass the lung tissue, pulmonary vessels and the air within the lungs [27].

In the resulting images of the grouping, the appearing of small structures that are not grouped, including juxtapleural nodules, is common. Juxtapleural nodules are the ones attached to the pleural surface. This segmentation problem is caused due to the fact that nodules have Hounsfield Units similar to the pleura [13]. In order to include these structures, a 3D morphological closing filter with twelve units in radius, by the ITK toolkit, was used to perform a binary dilation followed by an erosion [28]. As a result, it creates a binary mask with voxels. Inside the lungs, the value is one and outside the lungs, the value is zero. This mask is used to determine the volume of the segmented lung, i.e. the voxel number inside the lung. If the volume is close to the statistic average found in lung volumes (equal to 3,545,668 voxels), the mask will be applied in the original image and the segmented lung will be shown to the user. If the volume of the mask is of high variance (over 60 %), a new attempt to group voxels will be held. If the volume of the mask corresponds to the statistic average and if the user accepts the segmentation, the process ends. Otherwise, if the user does not accept the segmentation, the user must inform other seed points to restart the process.

#### 3D segmentation of internal structures of lungs

**Fig. 3:**
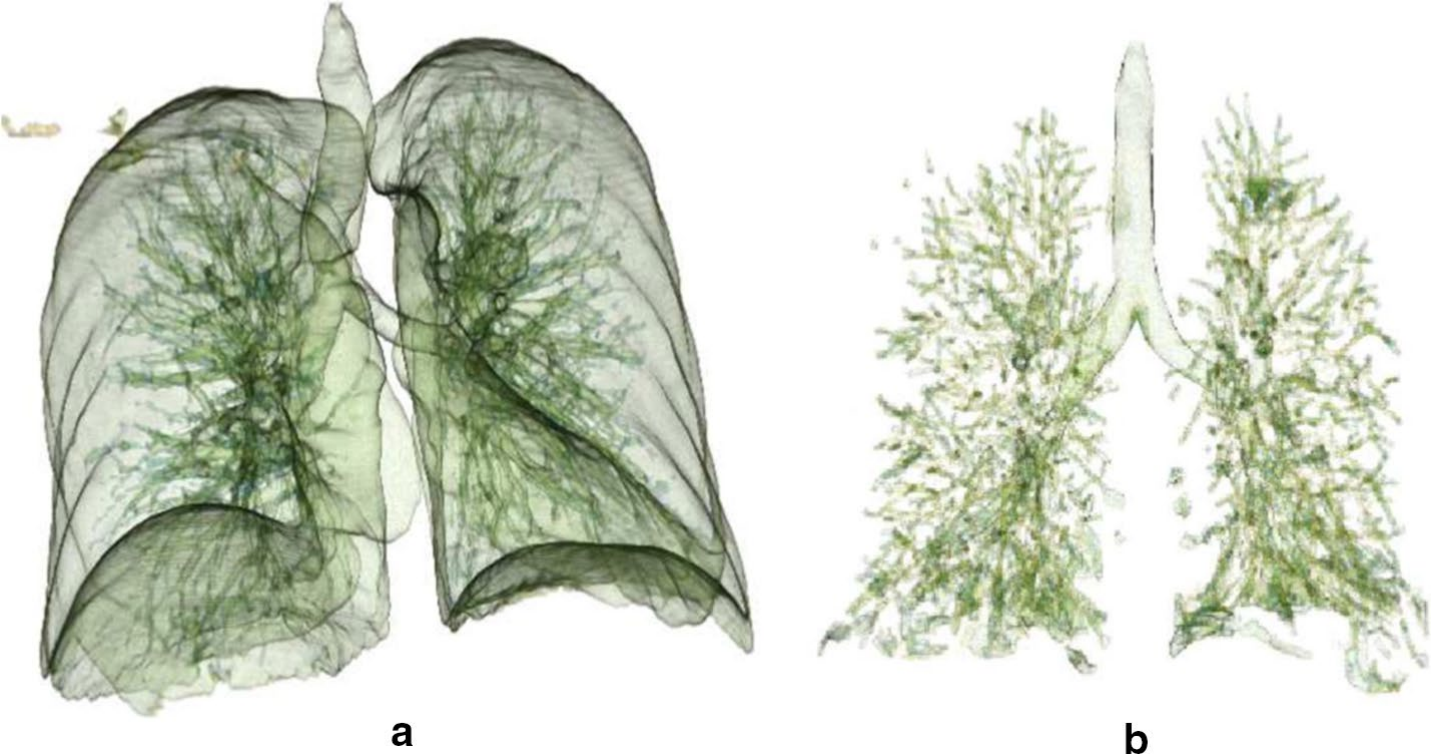
3D reconstruction: **a** of the lungs and **b** of lung structures segmented by the proposed method

After the segmentation process of the lungs, the segmentation of internal organs is performed. In this segmentation, the internal structures (e.g., trachea, bronchi and pulmonary vessels) are separated aiming to distinguish pulmonary nodules, in case there is any. In this part, the Watershed transform was used, proposed by Vincent and Soille [29, 30] and implemented by ITK Toolkit [26]. This method defines a function *f*(*x*, *y*, *z*) to group a set of voxels that are local minima. The proposed method used the function *f*(*x*, *y*, *z*) for calculating the magnitude of the gradient shown in Eqs. 1 and 2.

1 $$\begin{aligned} f(x,y,z) = \sqrt{\left( \frac{\partial J}{\partial x} \right) ^{2} + \left( \frac{\partial J}{\partial y}\right) ^{2} + \left( \frac{\partial J}{\partial z}\right) ^{2}}\end{aligned}$$

2 $$\begin{aligned} J_w = I \odot \left( \frac{\partial }{\partial _{w}} \odot G \right) \end{aligned}$$

where: *I* is the original 3D image and *G* is a 3D Gaussian function. Through this segmentation, it is possible to group the tissues that have similar intensities allowing lung structures to be separated, especially pulmonary nodules from other structures. Figure 3 shows an example of a reconstruction of the segmented lungs and of the pulmonary structures segmented by the Watershed transform with the function to calculate the gradient magnitude.

#### Detection of candidate pulmonary nodules

The diagnosis of lung cancer usually begins with the identification of an abnormality in radiological tests. These abnormalities are very variable and depend on their location and relationship with the bronchi and vessels. However, the most common radiological patterns are: collapse, consolidation (mass), pleural effusion and different combinations [31]. Initially, they are rounded, but, according to growth, they tend to lose this shape and take more irregular settings and ill-defined contours. The nodules can be classified into: small nodules, nodules attached to vessels (called juxtavascular), nodules attached to the wall of the lung (called juxtapleural) and ground-glass nodules [21].

**Fig. 4:**
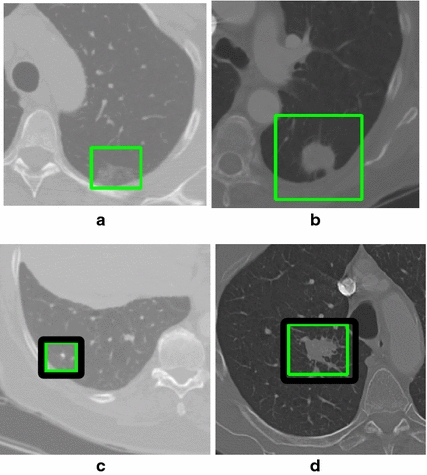
Examples of different types of lung nodules. **a** ground-glass nodule with irregular shape, **b** solid juxtapleural nodule in *ovoid shape*, **c** solid spherical nodule with 4 mm in diameter and **d** solid juxtavascular nodule in *ovoid shape*

Small nodules represent nodules with a diameter smaller than 5 mm. Juxtavascular nodules refer to nodules that are connected to blood vessels, while juxtapleural nodules refer to cases in which they are connected to the parenchyma wall or to the diaphragm. Ground-glass nodules refer to a type of nodule where the intensity value of the pixels are significantly lower than those of solid nodules [32]. Figure 4 shows examples of the following types of nodules: ground-glass nodules, juxtapleural nodules, small nodules and juxtavascular nodules.

To analyze the internal structures that have been segmented and separate the possible nodules, a rule-based classifier has been used. The first rule, referred to as Roundness was applied to the segmented structure aiming to detect spherical or semi-spherical objects. Its calculation is shown in Eq. 3. Whenever the Roundness exceeds a threshold, the segmented object is considered to be a non-nodule and disposed from later stages. The second rule, called Elongation, aims to detect cylindrical structures. Its calculation is shown in Eq. 4. Whenever the Elongation is less than a threshold, the object is considered to be a non-nodule.

The third rule is based on image texture by calculating the Energy. The Energy, calculated through the co-occurrence matrix, expresses the uniformity of texture on the image aiming to eliminate regions that do not contain nodules. The energy is calculated by Eq. 5 and if the measured value is less than a threshold, the object is not considered to be a nodule.

3 $$\begin{aligned}&(R1)&Roundness = \frac{A_n (r)}{a} < 8.3 \times 10^4\end{aligned}$$

4 $$\begin{aligned}&(R2)&Elongation = \frac{MP_{max}}{MP_{min}} < 6.8 \times 10^4\end{aligned}$$

5 $$\begin{aligned}&(R3)&Energy = \sqrt{\displaystyle \sum _{1}^{ns} \displaystyle \sum _{levels-1}^{i,j=0} P_{i,j}^2} < 3.3 \end{aligned}$$

where: $A_n$ is the area of a hypersphere (of radius *r*) that has the same nodule volume, *a* is the area of the nodule, $MP_ {max}$ is the largest main image moment $MP_{min}$ is the smallest main image moment, *ns* is the number of slices where the nodule appears, *levels* corresponds to the maximum intensity value in the gray scale of the image and $P_{i,j}$ is the histogram of co-occurrence of gray level of the image. More details about Roundness, Elongation and Energy, such as definitions and parameters can be found in Lehmann [28] and Hall-Beyer [33].

The rule-based classifier has been used to quickly remove some structures that are easily distinguishable as False Positives (e.g., bronchi, trachea, pulmonary vessels) so as to eliminate the influence of such structures on the subsequent stages. In this work, the rules were designed based on knowledge gained from radiologists and statistical study of the morphological characteristics, intensity and texture of lung nodules and non-nodules at CT scans available in LIDC-IDRI. The rules were defined with relatively tenuous criteria so that they are not specific to the data set used in this work.

#### Elimination of false positives

At this stage we will eliminate remaining false positives (FPs) while preserving true positives. In the context of CADe systems, the false positive term means lesions that are identified by the CAD algorithm, but are not nodules. Typical false positives were: vessels with sharp curvature, thick vessels with bifurcations, stains generated by respiratory or cardiac motion and scarring on the parenchymal tissue (parenchymal tissue).

Typically, FPs are removed by classification algorithms. For this, candidate nodules are segmented and their features are extracted. The job of the classifier is to determine boundaries for the separation of classes (i.e. nodules and non-nodules) based on the extracted features. The method of feature extraction of nodules used was the Histogram of Oriented Gradient (HOG) [34] of the Skimage library [35]. Skimage is a collection of algorithms for image processing and computer vision available free of charge and free of restrictions. The basic idea of this method is that the appearance and shape of objects present in images can be characterized by the distribution of the intensity and direction of the gradients of pixels.

The HOG subdivides the images into small regions (called cells) and for each pixel inside the cell its gradient is calculated through Eq. 6. Thereafter, for each cell it computes a histogram of the gradients. The gradient indicates the direction of maximum intensity variation near the pixel, catching contours, silhouettes and some information about the texture. To improve accuracy, the cells histograms are normalized by their grouping with the neighboring cells histograms. This grouping of cells is called a block and this normalization results in better invariance to the changes in lighting and shading.

6 $$\begin{aligned} \nabla f = \frac{\partial f }{\partial x} X + \frac{\partial f }{\partial y} Y \end{aligned}$$

where: $\frac{\partial f}{\partial x}$ is the gradient in the direction X and $\frac{\partial f}{\partial y}$ is the gradient in the direction Y.

The HOG was calculated for each slice of the object. Then a resulting histogram was generated by grouping the HOG of each slice. The result presented high dimensionality with feature vectors with dimensions between 77 and 2,380,848 for each candidate nodule. In this way, the Principal Component Analysis (PCA) was applied to reduce the dimensionality, so that data can be handled and stored more efficiently.

PCA is a mathematical method, proposed by Hotelling [36], which uses orthogonal transformation to convert a set of variables, possibly correlated, to a set of values of linearly uncorrelated variables called principal components. The reduction of the dimension of the data consists in obtaining the main components from the ordering of the extracted eigenvalues of the covariance matrix of the original data [37]. The method proposed by Thomas P Minka [38] allows the use of PCA by defining a minimum percentage of variance to be maintained without the need to determine in advance the number of components. With this, PCA was used keeping 80 % of the variance of the original data. After the PCA, the feature vector of candidate nodules had a dimension of 73. The PCA algorithm used in this study was implemented by the Sklearn library [39]. Sklearn is a simple and efficient open source tool for data mining and data analysis.

Finally, the Support Vector Machines (SVM) classifier is used to analyze the results of the PCA. The SVM is a technique based on the Statistical Learning Theory of the type supervised training [40], able to generalize problems of binary classification from a data set. Its operation is given through nonlinear functions (called kernels) that map input vectors in a high dimensional space (called feature space) [41]. In the proposed method the SVM classifier was chosen for elimination of False Positives because it provides the best results when compared to other classifiers. This comparison will be presented in the "Results and discussion" sections.

#### Compute the likelihood of malignancy

Once detected, the next stage is to determine the likelihood of malignancy of the nodules. Regarding the texture, nodules with ground-glass opacity are malignant in 59–73 % of cases, while solid nodules have a probability of 7–9 %. Regarding its shape, nodules of irregular shape have a higher likelihood of malignancy compared to round nodules. Calcification patterns of nodules are also useful to determine the malignancy. Calcification patterns for benign nodules have been described as: central, popcorn, solid and laminated. Benign nodules often have well-defined smooth edges. Spiculated nodules and nodules with irregular or lobular margins are more often malignant nodules.

According McNitt-Gray et al. [42] the probability of malignancy is related to the age of the patient, whether he is a smoker and the features of shape and appearance of nodules. These features are: calcification patterns, internal structure, margin, shape, texture and presence of lobulation and spiculation. Thus, the proposed CAD system will use the concepts of McNitt-Gray et al. to determine the likelihood of malignancy considering patients older than 60 years old and known to smoke. The likelihood of malignancy is divided into five degree: highly unlikely, moderately unlikely, indeterminate, moderately suspicious and highly suspicious. The use of five degree was suggested by experienced radiologists interviewed.

**Fig. 5:**
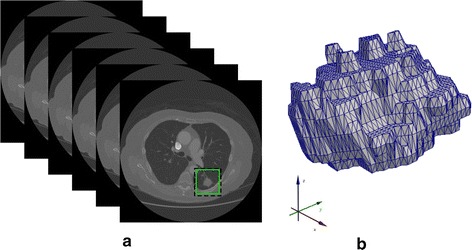
Examples of a nodule detected by the system. **a** A highlighted nodule in the various slices of the test, **b** 3D reconstruction of the nodule found

The radiologist should see the highlighted nodules and their 3D reconstruction, as shown in Fig. 5. For reconstruction, the VTK library was used [43]. As a result, radiologists must report seven features of texture, shape and appearance of nodules. These features are: calcification patterns, internal structure, Lobulation, Margin, Sphericity, Spiculation and Texture. The features and their respective values are shown in Table 1.

**Table 1:** Features of texture, shape and appearance of nodules and their values that radiologists should tell the system

Features	Values
Calcification	Popcorn, laminated, solid, non-central, central, and absent
Internal structure	Soft tissue, fluid, fat, and air
Lobulation	Marked, intermediate, and none
Margin	Poorly defined, intermediate, and sharp
Sphericity	Linear, ovoid, and round
Spiculation	Marked, intermediate, and none
Texture	Non-solid, Part solid/(mixed), and solid

With the values provided by radiologists, the system uses an SVM classifier, trained previously, to determine the likelihood of malignancy. The SVM classifier was chosen because it provides the best results when compared to other classifiers tested. Based on the likelihood of malignancy, the radiologist may have more information to take the decision on the treatment and monitoring of patients.

## Results and discussion

Lung cancer is responsible for over 1.59 million deaths each year. CAD systems are being developed to assist radiologists in the detection and diagnosis in order to decrease this rate. These systems must provide acceleration in the diagnosis, reducing errors and improving the quantitative evaluation. Currently, the main criteria used to evaluate the detection of CAD systems (CADe) are: false positive (FP) rate and sensitivity. FP is when the system presents positively to a nodule when in reality it does not exist. The sensitivity (S) is the relation between true positives and the false negative, given by Eq. 7.

7 $$\begin{aligned} \texttt {S} = \frac{TP}{(TP + FN)} \end{aligned}$$

where: *S* is the sensitivity, *TP* is the true positive rate and *FN* is the false negative rate. TP represents the results that the system presented positively to a sample that actually had the disease. FN represents the negative results when the sample had the disease.

The Receiver Operator Characteristics (ROC) is the performance measure most widely used to evaluate the diagnosis of CAD systems (CADx). The ROC provides an index related to the accuracy and effectiveness through the relationship between the probability of true positives and the probability of false positives. The ROC curve is created by plotting the true positive (TP) rate on the Y axis, and the false positive (FP) rate on the X axis at various threshold settings. After the computation of the ROC curve, the area under the curve ($A_z$) should be calculated using the Eq. 8. Sklearn library [39] was used to generate the ROC curve and calculate the *Az*.

8 $$\begin{aligned} A_z = \int ^{\infty }_{-\infty } \texttt {TPR}(T) \texttt {FPR'}(T) dT \end{aligned}$$

where: TPR is true positive rate and FPR is false positive rate.

To validate the CAD system 420 CT scans were used. Computation time of the proposed system was approximately 12 min per case using a notebook with Intel Core i7-4500U CPU 1.80 GHz × 4. The segmentation stage had an accuracy of 97 % and is not effective in cases of severe pathologies, which alter the opacity of the lung outlines.

### Validate of classifier

The method to validate the ability of generalization of the classifier was the 10-fold Cross Validation and Leave-one-out. The effectiveness of the SVM is verified by comparing with FLD (Fisher’s Linear Discriminant) and Gaussian Naive Bayes. The SVM used was the C-Support Vector Classification (SVC) with radial kernel implemented by the Sklearn library. SVC is one implementation of SVM with multiclass support that performs a one-vs-one approach [44]. If *k* is the number of classes, then $k(k-1)/2$ classifiers are constructed and each one trains data from two classes. When training an SVM with the Radial Basis Function kernel, two parameters must be considered: C and gamma. The parameters used were $C = 5$ and

9 $$\begin{aligned} gamma = \frac{1}{n} = {\left\{ \begin{array}{ll} \frac{1}{77} &{}\text{ in } \text{ detection, } \\ \frac{1}{7} &{}\text{ in } \text{ diagnosis }. \end{array}\right. } \end{aligned}$$

where: *n* is the number of features. Other values were tested but these showed better results.

Fisher’s Linear Discriminant (FLD) is a supervised classifier that projects high-dimensional data onto one-dimensional space for classification [45]. The FLD used was implemented by the Sklearn library with Singular Value Decomposition solver. Naive Bayes methods is a supervised classifier based on applying Bayes’ theorem with MAP (maximum a posteriori) estimation [46]. The Naive Baye used was the Gaussian Naive Bayes implemented by Sklearn. Following the results of validation tests will be presented.

#### 10-fold cross validation

In the 10-fold Cross Validation method the original database is randomly separated in *k* mutually exclusive subsets and of the same size. These *K* subsets, $K-1$ are used to train the classifier and 1 subset is used for validation tests. This procedure is repeated until all *k* subsets are used for the tests. The result of this process is the average of performance in all tests. The aim of repeating multiple times is to increase the reliability of the estimate of the accuracy of the classifier. In the tests performed, we used $k = 10$. This method is commonly used in validation of CAD systems [7–10]. The results are shown in Table 2.

**Table 2:** Performance comparison of classifiers for detection and diagnosis using 10-fold cross validation

Classifier	Sensitivity (%)	FP	$A_z$
FLD	89.2	6.89	0.91, 0.81, 0.69, 0.70, 0.81
Naive Bayes	93.9	7.03	0.89, 0.82, 0.67, 0.70, 0.83
SVM	94.4	7.04	0.91, 0.80, 0.72, 0.67, 0.83

As result, the CAD system with SVM performs better. The sensitivity found for detection was of 94.4 % with a FP rate of 7.04 per case. The area values of the ROC curve found with SVM were between 0.91 for the nodules with highly unlikely malignancy, 0.80 for nodules with moderately unlikely malignancy, 0.72 for nodules with indeterminate malignancy, 0.67 for the nodules suspected of moderately malignancy and 0.83 for highly suspected malignant nodules.

#### Leave-one-out validation

The Leave-one-out Validation is a statistical technique used to determine, during training, the generalization capability of classifiers [40]. The dataset should be divided randomly into two distinct sets, one for training (used to train) and one for validation (used to validate). This method also is used in validation of CAD systems [47, 48]. In the tests we used: the training subset containing 294 CT scans (with 215 cancerous and 79 non-cancerous) and the validation subset containing 126 CT scans (with 89 cancerous and 37 non-cancerous). The effectiveness of the SVM is verified by comparing with FLD (Fisher’s Linear Discriminant) and Gaussian Naive Bayes, both implemented by the Sklearn library. The results are shown in Table 3.

**Table 3:** Performance comparison of classifiers for detection and diagnosis using leave-one-out validation

Classifier	Sensitivity ( %)	FP	$A_z$
FLD	88.99	7.47	0.93, 0.79, 0.68, 0.72, 0.80
Naive Bayes	90.47	7.44	0.92, 0.80, 0.66, 0.70, 0.85
SVM	93.9	7.21	0.93, 0.78, 0.69, 0.67, 0.85

Through the Table 3, it can be inferred that the performance of the SVM is the best. The sensitivity found for detection was of 93.9 % with a FP rate of 7.21 per case. The area values of the ROC curve found with SVM were between 0.93 for the nodules with highly unlikely malignancy, 0.78 for nodules with moderately unlikely malignancy, 0.69 for nodules with indeterminate malignancy, 0.67 for the nodules suspected of moderately malignancy and 0.85 for highly suspected malignant nodules.

### Comparing the performance

A relative comparison of our detection method using SVM with other mentioned in the literature was performed and shown in Table 4. In this Table, each line represents a CADe system for the detection of pulmonary nodules on CT images. For each system it presents the sensitivity obtained, numbers of false positives and number of nodules used for validation. Based on this comparison, it can be inferred that the performance of the proposed system is among the best in sensitivity and has been tested with a larger amount of nodules.

**Table 4:** Performance comparison of pulmonary nodule detection methods by sensitivity, FP and the number of nodules used in the validation

Methods	Sensitivity (%)	FP	N of nodules
Armato et al. [6]	70	9.6/case	187
Suzuki et al. [7]	80.3	4.8/case	121
Messay and Rogers [8]	82.66	3/case	143
Tan et al. [9]	87.5	4/case	574
Cascio et al. [10]	97	6.1/case	148
Teramoto e Fujita [11]	80	4.2/case	103
Han et al. [12]	82.7	4/scan	490
Our method	94.4 %	7.04/case	1109

**Fig. 6:**
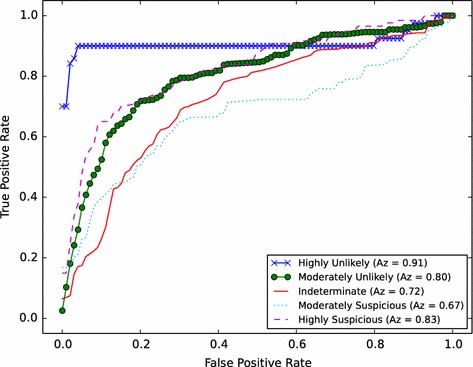
ROC curve for the distinction of classes: Highly Unlikely, Moderately Unlikely, Indeterminate, Moderately Suspicious and Highly Suspicious, obtained in the 10-fold Cross Validation with SVM

The Fig. 6 shows the results obtained by the system, with SVM method, in the diagnosis of malignancy in terms of ROC curves. A literature review was conducted to identify the results of other CADx systems for the diagnosis of lung cancer and compare with the proposed CADx system. A summary of the results obtained by other CADx systems and the proposed method is shown in Table 5.

**Table 5:** Performance comparison of diagnostic methods of pulmonary nodules by ROC curve

Methods	$A_z$	Classes
Shah et al. [15]	0.92	Malignant and benign
Way et al. [16]	0.857	Malignant and benign
Suzuki et al. [17]	0.88	Malignant and benign
Lee et al. [18]	0.889	Malignant and benign
Orozco et al. [19]	0.805	Malignant and benign
Our method	0.91, 0.80, 0.72, 0.67 and 0.83	Likelihood of malignancy

In Table 5, each row represents a published method followed by the area under the ROC curve obtained by the system and the type of classification performed. Based on this comparison, it can be inferred that the performance of our system is at the same level of the results shown in the mentioned documents. However, our system presents as advantage the diagnosis based in likelihood of malignancy through the subdivision into five degree, allowing more aid in the decision making by radiologists.

Accordingly, we believe that our system is clinically useful for the detection and diagnosis of pulmonary nodules, because it performed well in the detection, in the diagnosis and it has a good level of automation. The experimental results on the set of independent data show the generalization of the proposed method. However, the system does not detect lung nodules smaller than 3 mm and should not be used in cases in which there is the presence of severe pathologies, which alter the opacity of lung outlines.

## Conclusion

A new CAD system has been proposed for the detection and diagnosis of pulmonary nodules in CT images of the chest, grouping in a single system both identification and characterization of nodules. For this, the use of Histogram of Oriented Gradient (HOG) was proposed for characterization of nodules and the use of the Watershed technique to segment lung internal structures to separate the possible nodules from other structures. Besides, a rule-based classifier and a Support Vector Machine (SVM) have been used to eliminate false positives.

The segmentation method achieved an accuracy of 97 %, though not being effective in cases where severe pathologies occurred, altering the opacity of lung outlines. The detection system showed a sensitivity of 94.4 % with 7.04 false positive per case for the detection of nodules with diameters between 3 mm and 30 mm and of different types (isolated, juxtapleural, juxtavascular and ground-glass nodules). For the diagnosis of malignancy, our system presented ROC curves with areas of: 0.91 for nodules highly unlikely of being malignant, 0.80 for nodules moderately unlikely of being malignant, 0.72 for nodules with indeterminate malignancy, 0.67 for nodules moderately suspicious of being malignant and 0.83 for nodules highly suspicious of being malignant. The diagnosis based on likelihood of malignancy enables greater aid in the decision making made by radiologists.

From our preliminary results, we believe that our system is promising for clinical applications assisting radiologists in the detection and diagnosis of lung cancer. However, we are still working to enable the segmentation of the lungs of patients with diseases that alter the opacity and outlines of lungs and to automatically generate seed points. Image processing techniques are being analyzed in order to generate the characteristics of nodules (Calcification patterns, internal structure, Lobulation, Margin, Sphericity, Spiculation and Texture) directly from the images allowing greater automation of the system. As future work, we plan to conduct a clinical trial of the proposed method and verify their performance in a real environment, analyze other method of feature extraction of nodules and conduct another study to find the optimal values of SVM classifier.
